# Risk estimation of the SARS-CoV-2 acute respiratory disease outbreak outside China

**DOI:** 10.1186/s12976-020-00127-6

**Published:** 2020-06-05

**Authors:** Soyoung Kim, Sunhwa Choi, Youngsuk Ko, Moran Ki, Eunok Jung

**Affiliations:** 1 Department of Mathematics, Konkuk University, 120 Neungdong-ro, Gwangjin-gu, Seoul, 05029 South Korea; 2 Department of Cancer Control and Population Health, Graduate School of Cancer Science and Policy, National Cancer Center, 323 Ilsan-ro, Ilsandong-gu, Goyang, 10408 South Korea

**Keywords:** SARS-CoV-2, COVID-19, Risk estimation, Mathematical model, Stochastic simulation, Reproductive number

## Abstract

**Background:**

On December 31, 2019, the World Health Organization was alerted to the occurrence of cases of pneumonia in Wuhan, Hubei Province, China, that were caused by an unknown virus, which was later identified as a coronavirus and named the severe acute respiratory syndrome coronavirus 2 (SARS-CoV-2). We aimed to estimate the reproductive number of SARS-CoV-2 in the Hubei Province and evaluate the risk of an acute respiratory coronavirus disease (COVID-19) outbreak outside China by using a mathematical model and stochastic simulations.

**Results:**

We constructed a mathematical model of SARS-CoV-2 transmission dynamics, estimated the rate of transmission, and calculated the reproductive number in Hubei Province by using case-report data from January 11 to February 6, 2020. The possible number of secondary cases outside China was estimated by stochastic simulations in various scenarios of reductions in the duration to quarantine and rate of transmission. The rate of transmission was estimated as 0.8238 (95% confidence interval [CI] 0.8095–0.8382), and the basic reproductive number as 4.1192 (95% CI 4.0473–4.1912). Assuming the same rate of transmission as in Hubei Province, the possibility of no local transmission is 54.9% with a 24-h quarantine strategy, and the possibility of more than 20 local transmission cases is 7% outside of China.

**Conclusion:**

The reproductive number for SARS-CoV-2 transmission dynamics is significantly higher compared to that of the previous SARS epidemic in China. This implies that human-to-human transmission is a significant factor for contagion in Hubei Province. Results of the stochastic simulation emphasize the role of quarantine implementation, which is critical to prevent and control the SARS-CoV-2 outbreak outside China.

## Background

A novel coronavirus – the severe acute respiratory syndrome coronavirus 2 (SARS-CoV-2) – emerged in Wuhan, Hubei Province, China in late December 2019. On January 30, 2020, the World Health Organization (WHO) declared a Public Health Emergency of International Concern (PHEIC) [1], and on January 31, 2020, the number of coronavirus disease (COVID-19) cases exceeded that of SARS, with a reported 8096 people infected worldwide [2]. As of February 6, 2020, there were 28,018 confirmed cases, including 563 deaths, of COVID-19 reported in China. Moreover, 325 confirmed cases of SARS-CoV-2 infection have been reported from outside China across 27 countries, including Japan, Thailand, Singapore, South Korea, Hong Kong, Australia, Germany, the United States, Taiwan, Macau, and Vietnam [3].

The WHO confirmed the possibility of human-to-human transmission. Therefore, the epidemiological link, such as close contact tracing of exported/imported COVID-19 patients, is among the most important features for the prevention and control of the COVID-19 outbreak. In most of the developed countries, such as the United States, Europe, Japan, and South Korea, there is a well-established infectious disease prevention and quarantine policy even in the early stages of disease spread. However, the international travels of SARS-CoV-2-infected patients have threatened public health in other countries. Furthermore, it is worthwhile to measure the potential risk of SARS-CoV-2 transmission outside China when the quarantining of symptomatic infected individuals is delayed.

In this study, we used stochastic simulations to estimate the expected number of COVID-19 patients and epidemic duration in various scenarios of quarantine and reduction in the transmission rate. The potential size of the epidemic outside of China, especially in developed countries with a well-established public health infrastructure, is estimated by assuming similar transmission probabilities in the early disease stages as that in Hubei Province, as well as by considering the potential impact of various social and personal nonpharmaceutical interventions.

## Methods

### Data sources

Data were obtained from the 2019-nCoV Global Cases by the Johns Hopkins Center for Systems Science and Engineering [4] and Novel Coronavirus (2019-nCoV) situation reports issued by the WHO [5] (last retrieved on February 7, 2020). All reported data were from confirmed COVID-19 cases.

### Mathematical model

**Fig. 1:**
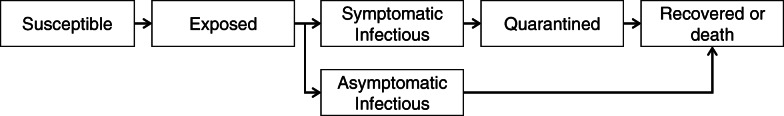
Schematic diagram of SARS-CoV-2 transmission dynamics

We constructed a dynamic model of SARS-CoV-2 transmission on the basis of a deterministic compartment model. The population was classified into six classes: susceptible (*S*), exposed (*E*), symptomatic infectious (*I*), asymptomatic infectious (*A*), quarantined (*Q*), and removed individuals (*R*). Susceptible individuals are exposed to the SARS-CoV-2 on close contact with infectious individuals or respiratory droplets that are generated when a patient coughs [6]. After the incubation period, the exposed individuals develop either symptomatic or asymptomatic infections. Asymptomatic individuals recover after the infectious period. However, symptomatic individuals who are infected are quarantined, and patients who are quarantined will recover or die. Figure 1 describes a schematic diagram of the transmission dynamics of SARS-CoV-2:

The transmission dynamics of SARS-CoV-2 acute respiratory disease are described by six ordinary differential equations (ODEs) as follows:

$$ {\displaystyle \begin{array}{c}\frac{dS}{dt}=-\beta \frac{\left(1-q\right)E+I+\left(1-\delta \right)A}{N}S,\\ {}\frac{dE}{dt}=\beta \frac{\left(1-q\right)E+I+\left(1-\delta \right)A}{N}S-\kappa E,\\ {}\frac{dI}{dt}=\left(1-p\right)\kappa E-\alpha I,\\ {}\frac{dA}{dt}= p\kappa E-{\gamma}_AA,\\ {}\ \frac{dQ}{dt}=\alpha I-{\gamma}_QQ,\\ {}\ \frac{dR}{dt}={\gamma}_AA+{\gamma}_QQ,\end{array}} $$

where *N* = *S* + *E* + *I* + *A* + *Q* + *R*.

The parameter *β* represents the transmission rate. In recent studies, the WHO and Chinese public health authorities have reported the possibility of transmission from exposed and asymptomatic infectious individuals. Because exposed and asymptomatic individuals do not have symptoms, they have lower transmissibility than that of symptomatic infectious individuals. The transmission-reduction factors of exposed and asymptomatic individuals are denoted by *q* and *δ*, respectively. As there is inadequate epidemiological evidence, such as the proportion of asymptomatic infections and transmissibility of exposed and asymptomatic infectious individuals, it is assumed that transmission of exposed and asymptomatic infectious individuals can be ignored. The parameter *κ* indicates the rate of progression from exposed to infectious individuals, and 1/*κ* represents the average incubation period. A proportion *p* (0 ≤ *p* ≤ 1) of newly infectious individuals become asymptomatic; thus 1 − *p* represents the proportion of individuals with symptomatic infectious. The parameter *α* represents the quarantine rate of symptomatic infectious individuals and 1/*α* indicates the average duration from symptom onset to quarantine. The parameter *γ*_*A*_ represents the recovery rate of asymptomatic individuals and 1/*γ*_*A*_ is the average recovery period of asymptomatic individuals. The parameter *γ*_*Q*_ indicates the removal rate of quarantined individuals and 1/*γ*_*Q*_ represents the average duration from quarantine to recovery or death.

The parametric values used in our model are listed in Table 1.

**Table 1:** Parameters of the SARS-CoV-2 transmission model

Symbol	Description	Value	Reference
*β*	Transmission rate	0.8238	datafitted
*q*	Transmission reduction factor for exposed individuals	1	Assumed
*δ*	Transmission reduction factor for asymptomatic infectious individuals	1	Assumed
*κ*	Progression rate from exposed to infectious individuals	1/5.2	[7]
*p*	Proportion of exposed individuals who become asymptomatic infectious	0	Assumed
*α*	Quarantine rate	1/5	[8]
*γ*_*A*_	Recovery rate for asymptomatic individuals	1/10	Assumed
*γ*_*Q*_	Removal rate for quarantined individuals	1/20	Assumed

The reproductive number, denoted by $\mathbf{\mathcal{R}}$, represents the average number of secondary cases generated by a single primary patient over its infectious period. In our model, the reproductive number, $\mathbf{\mathcal{R}},$ can be derived as follows by using the next generation method introduced by van den Driessche [9].

$$ \mathcal{R}=\beta \left(\frac{1-q}{k}+\frac{1-p}{\alpha }+\frac{p\left(1-\delta \right)}{\gamma}\right) $$

The reproductive number indicates the threshold for disease spread. If $\mathbf{\mathcal{R}}$ exceeds 1, an infectious individual transmits the disease to more than one individual and, eventually, the number of cases keeps increasing. On the other hand, if $\mathbf{\mathcal{R}}$ is less than 1, the number of cases continues to decrease, and the disease will die out. Using this property, we can identify an intervention strategy, which can reduce the reproductive number to less than 1.

### Model calibration

The transmission rate was estimated from the number of cases that was confirmed per day in Hubei Province from January 11 to February 6, 2020, and then best-fitted to the model by using the least squares method. All confirmed cases were assumed to be quarantined and incapable of spreading the disease. Our model was programmed in Matlab 2018b. The least squares fitting optimization tool, *lsqcurvefit*, was used to estimate the best-fitted transmission rate. For uncertainty analysis, the transmission rate was estimated by using the data generated from the Poisson distributions, with the mean equal to the observed data. Mean, standard deviation, and 95% confidence intervals (CI) were calculated from 1,000 sample datasets. In addition, we conducted a sensitivity analysis by varying the parameters over a range of possible values to establish which of the parameters had the greatest effect on the reproductive number.

### Stochastic simulation

Stochastic simulation was implemented to estimate the risk of an COVID-19 outbreak in a country outside China, due to the arrival of an exposed individual. According to the various scenarios of transmission reduction and the duration from symptom onset to quarantine, we calculated the average number of local transmission cases, the probability of an outbreak, and the epidemic duration. The Gillespie algorithm, which is an affordable method to observe individual random events, was adapted as the stochastic simulation method [10]. Table 2 lists the possible individual level events in stochastic simulation and their propensities, which emerge from the mathematical model. In the Gillespie algorithm, the probability of the event occurrence is proportional to its propensity, whereas the time interval between a prior and posterior event is proportional to the sum of every propensity in the prior event.

**Table 2:** propensity of individual events

Event	Propensity
*S* → *E*	$\beta \frac{\left(1-q\right)E+I+\left(1-\delta \right)A}{N}S$
*E* → *I*	(1 − *p*)*κE*
*E* → *A*	*pκE*
*I* → *Q*	*αI*
*Q* → *R*	*γ*_*Q*_*Q*
*A* → *R*	*γ*_*A*_*A*

## Results

### SARS-CoV-2 transmission dynamics in Hubei Province

**Fig. 2:**
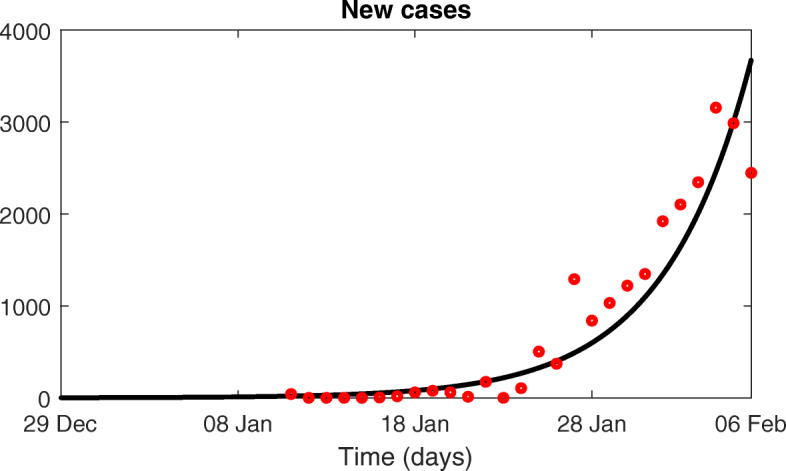
Daily reported data of confirmed cases in Hubei province and best-fitted model curve

The rate of transmission in Hubei Province is estimated to be 0.8238 (95% CI: 0.8095–0.8382) and the reproductive number was calculated as 4.1192 (95% CI: 4.0473–4.1912). Figure 2 presents the data-fitted results from December 29, 2019 to February 6, 2020. The red circles indicate the daily reported data of confirmed cases in Hubei Province, and the black curve represents the best-fitted model curve. Our model initially comprised four symptomatic infectious individuals [7]; furthermore, confirmed cases reported from January 11, 2020 were used for data-fitting.

### Risk estimation of COVID-19 outbreak outside China

The COVID-19 outbreak in Wuhan has spread in China as well as to other countries. The risk of outbreak is investigated as the entry of an exposed individual to a country outside of China. We assumed there would be a lower rate of transmission and shorter duration from symptom onset to quarantine outside China. For the purpose of this research, we considered ten reductions in transmission (from 0 to 90% at 10% intervals) and three durations from symptom onset to quarantine (24, 36, and 48 h). We investigated 30 different scenarios based on changes in the quarantine rate and transmission reduction. Among those 30 scenarios, 9 selected scenarios are listed in Table 1, including the number of individuals with local transmissions, the epidemic duration, the maximum prevalence, and the probability of the estimated total cases from 2,000 realizations. Table 3 lists the results for three reductions in transmission (0, 30, and 60%). We defined prevalence as the total number of exposed, infectious, quarantined hosts, and the epidemic duration as the interval from symptom onset of the index case to quarantine onset of the last case. Every result, except for probabilities, is presented as the mean, lower bound (2.5th percentile), and upper bound (97.5th percentile) values of the trials, respectively. The results from the simulation of other cases of reduction in the rate of transmission are provided in the Supplementary Table.

**Table 3:** Estimated number of local transmission cases and epidemic duration of SARS-CoV-2 transmission outside of China

Transmission reduction	0%			30%			60%		
Period from symptom onset to quarantine (hours)	24 h	36 h	48 h	24 h	36 h	48 h	24 h	36 h	48 h
Local transmission cases, mean (Lb, Ub)	4.98, (0, 48)	192.86, (0, 1824)	7804.75, (0, 40,180)	1.44, (0, 12)	7.27, (0, 54)	75.13, (0, 714)	0.49, (0, 4)	1.54, (0, 9)	3.49, (0, 21)
Maximum daily prevalence, mean (Lb, Ub)	3.99, (1, 25)	109.69, (1, 1057)	6013.4, (1, 31,416)	2.16, (1, 10)	5.15, (1, 27)	38.32, (1, 354)	1.45, (1, 4)	2.21, (1, 8)	3.38, (1, 13)
Epidemic duration (days), mean (Lb, Ub)	15.87, (0.55, 81.27)	32.79, (0.77, 100.1)	46.85, (0.95, 100.01)	10.92, (0.6, 44.15)	17.73, (0.73, 94.17)	31.01, (0.87, 100.17)	8.49, (0.65, 31.17)	10.86, (0.66, 40.63)	13.34, (0.83, 52.91)
Probability of local transmission cases									
0 (%)	54.9	23.7	8.5	63.85	32.95	14.05	75.85	49.15	28.05
20 or more (%)	7	29.55	59.8	1	8.8	30.55	0	0.45	2.9
40 or more (%)	3.2	24.35	53.65	0	3.85	23.2	0	0	0.35

(Lb, Ub) = (lowerbound, upperbound)

We found a high deviation in the number of local transmission cases of infection (maximum prevalence) among the trials, as the upper bound was approximately more than five times larger than the mean in every scenario.

**Fig. 3:**
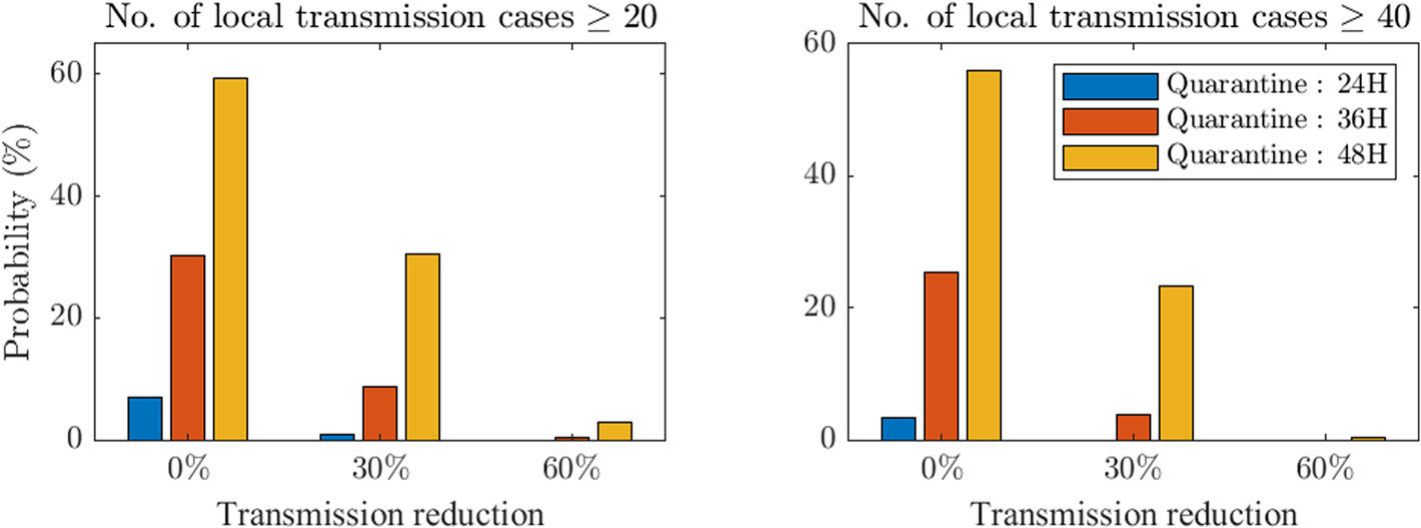
The probability of local transmission cases according to the nine scenarios

Figure 3 depicts the probability (%) of a certain number of local transmission cases being exceeded in relation to the reduction in the rate of transmission and duration from symptom onset to quarantine. When quarantine can be instituted within 24 h, the probability that the number of local transmission cases exceed 40 is 3.2% (scenario with a 0% reduction in the rate of transmission).

## Discussion

We aimed to present an initial perception of the transmission dynamics of SARS-CoV-2 in Hubei Province, China, and to enable a quantification of the potential risk of SARS-CoV-2 transmission outside China, especially when an infected patient from China arrives in developed countries. We used confirmed data from the clusters of COVID-19 cases that occurred from January 11 to February 6, 2020 in Hubei Province, China.

Many researchers have warned the possibility of outbreak outside of China. Thompson calculated the potential transmission when the patients arrived in other countries [11]. The risk of outbreak according to the imported cases, the connectivity of the country with China, and the efficacy of control measures is estimated [12]. In addition, studies have been conducted on the impact of international travel and border control measures on the spread outside China (global spread) [13, 14]. In this study, we used mathematical modeling and stochastic simulation to estimate the reproductive number and analyze interventional measures. The reproductive number for SARS-CoV-2 transmission dynamics is estimated approximately as 4, which is significantly higher compared to that of the previous SARS epidemic in China [15]. This implies that human-to-human transmission is significant in Hubei Province. Unless additional interventional strategies are implemented, there will be a significant increase in the daily incidence of new cases. However, several control policies are being implemented in China, such as public education on disease prevention and environmental hygiene. It is possible that the transmission rate and reproductive number have been decreased such that the number of new cases will be less than the number in the model prediction.

The reproductive number derived in this work facilitates the evaluation of control measures, such as quarantine or transmission-intervention strategy. To reduce the reproductive number to less than 1 with a single intervention measure, we need to either reduce the rate of transmission by more than 75.72% or shorten the duration from infection to quarantine to less than 29.1 h. From January 31, 2020 onward, a real-time confirmatory procedure has been instituted whereby the time to laboratory confirmation can be reduced to 6 h [16]. Moreover, a rapid diagnosis test kit has been provisionally approved, and this new diagnostic method has become available since February 7, 2020, which could possibly enable faster diagnosis and quarantine [17]. If two strategies are implemented simultaneously, the requirement for each intervention strategy will be reduced when compared to the requirement for a single-intervention strategy. For example, if the rate of transmission is reduced by 10%, the quarantine from symptom onset needs to be implemented within 32.37 h.

In this study, we assumed that the probability of transmission from exposed individuals was zero because of insufficient epidemiological evidence that could be applied in the model. However, if we assume that exposed individuals have a 20% rate of transmission compared to symptomatic individuals, the reproductive number would be reduced to 3.4796. Furthermore, our model assumes that quarantined individuals would not transmit the disease. Ignoring this assumption would result in a higher reproductive number.

This study was conducted with an aim to understand the requirements of sufficient preparedness to counter the spread of SARS-CoV-2 infection outside China; thus, we assumed the highest rate of transmission. Moreover, a stochastic simulation was conducted to measure the probability of an outbreak and its size when a person with infection exposure from China enters another country. If a symptomatic infectious individual is quarantined within 24 h of symptom onset, we can expect four cases of local transmission to occur. The epidemic duration would be approximately 17 days under the same rate of transmission as in Hubei Province. Therefore, the number of individuals with local transmission would be significantly decreased if patients are isolated within 24 h of symptom onset.

The WHO has warned of the possibility of global spread to other countries, and public health authorities in these countries are expected to develop preparedness strategies, such as thorough campaigns for enhancing personal hygiene, screening the national borders, and quarantine of individuals with suspected infection or exposure, to respond to disease spread. In South Korea, the first case of COVID-19 was reported on January 20, 2020, and a total of 24 confirmed cases, who are mostly individuals who returned from the Chinese city of Wuhan, have been reported as of February 7, 2020. Among these cases, nine were identified as local transmissions (human-to-human transmission) arising from contact with patients from China or with a secondary infection-transmitted person in South Korea [18]. Given that the average duration from symptom onset to quarantine is approximately 24 h in South Korea, the rate of transmission is considered to be approximately 50% lower than that in Hubei province. In the United States, one case of local transmission has been identified from among 11 confirmed cases with the SARS-CoV-2 infection [19]. The local transmission of infected case per import case is 1/11, and this implies that the rate of transmission is reduced by approximately 90% compared to that in the Hubei Province, if a patient can be quarantined within 24 h after symptom onset.

## Conclusion

Results from our risk estimation of the COVID-19 outbreak outside China accentuate the importance of quarantine control, such as through specific guidelines with regard to visitors who have traveled from China. The results of our stochastic simulation emphasize that rapid quarantine before the rate of community transmission increases is crucial to prevent a COVID-19 outbreak outside of China. Even if the rate of transmission is assumed to be similar to that in Hubei Province, the probability of no local transmission is approximately 54.9% with the implementation of a 24-h quarantine strategy. In addition, there is only a 7% chance of seeing more than 20 cases of local transmission. Thus, quarantine is implicated as a significantly important measure to minimize community transmission.

This study has some limitations. First, our model was developed with limited confirmed data from the Hubei Province of China; given that the outbreak has not ended, the model dynamics could change as new confirmed cases are added. Second, more detailed patient information, particularly the dates of symptom onset and exposure, were unavailable at the time of analysis. Third, this study was conducted on the basis of data from confirmed COVID-19 patients with symptomatic onset who underwent testing. However, some cases of asymptomatic infection have been identified [20, 21] and, given the availability of options for assessment at present, it is difficult to explicitly estimate the possibility of transmission or the number of asymptomatic infections based on the observational empirical data. Nonetheless, even a model with limited data, such as the one in this study, can allow an early assessment of the transmission dynamics of the SARS-CoV-2 epidemic in the Hubei Province, China.

This study describes an outbreak of the SARS-CoV-2 that originated in the Hubei Province and estimates the reproductive number. The epidemic has spread significantly due to failures in early diagnosis and rapid quarantine. In view of the difficulty in controlling this outbreak, our results lead us to strongly recommend that an infected person be quarantined as soon as possible after diagnosis to reduce the possibility of SARS-CoV-2 transmission outside China.

## Supplementary information

**Additional file 1.**

## Data Availability

Collected datasets are publicly available from reference [8, 9].

## Abbreviations

SARS-CoV-2: Severe acute respiratory syndrome coronavirus 2
COVID-19: Coronavirus disease
CI: Confidence interval
PHEIC: Public Health Emergency of International Concern
WHO: World Health Organization
ODE: Ordinary differential equation
