# Supplementary material for: Risk estimation of the SARS-CoV-2 acute respiratory disease outbreak outside China
Source: Theor Biol Med Model. 2020 Jun 5;17:9. doi: 10.1186/s12976-020-00127-6 (PMC7272210; doi:10.1186/s12976-020-00127-6)
Supplement: Supplementary file 1 — Additional file 1. [file 12976_2020_127_MOESM1_ESM.docx]

**Supplementary appendix**

**Risk estimation of the SARS-CoV-2 acute respiratory disease outbreak outside China**

Soyoung Kim*^1^, Sunhwa Choi*^2^, Youngsuk Ko^1^, Moran Ki^2^, Eunok Jung^1^

^1^ Department of mathematics, Konkuk University, Seoul, South Korea

^2^ Department of Cancer Control and Population Health, Graduate School of Cancer Science and Policy, National Cancer Center, Goyang, South Korea

*Contributed equally as co-first authors

**Correspondence to** Eunok Jung

Department of mathematics, Konkuk University

120 Neungdong-ro, Gwangjin-gu, Seoul 05029, South Korea

junge@konkuk.ac.kr

+82-10-9973-4163

**Uncertainty analysis of estimated parameters**

The parametric bootstrap (1, 2) is used for uncertainty analysis of the estimated transmission rate. The transmission rate is estimated by using the resampling data from the Poisson distributions with mean equal to the observed data. It is assumed that the observed data are Poisson distributed at a fixed time. With 1000 times sampling, the mean, standard deviation (S.D.), and 95% confidence intervals (CI) are calculated. The parametric bootstrap result provides the reliability of estimated model parameter.

Figure 1 shows the distributions of bootstrapping estimations for the transmission rate, $\beta$. The means, S.D., and 95% CI are listed in Table 3. A comparison between the bootstrap estimations and the best-fitted transmission rate shows the reliability of estimated parameter.

**Figure 1 Bootstrap distributions with 1000 resampling simulations**

**Table 3 Estimated parameter values, corresponding mean, standard deviation (SD), and 95% CI**

| Parameter | Estimated value | Mean | S.D. | 95% CI |
| --- | --- | --- | --- | --- |
| $\beta$ | 0.8238 | 0.8239 | 9.3633e-04 | (0.8220, 0.8257) |

**Parameter sensitivity**

In order to find the parameters that have the greatest impact on the reproductive number, sensitivity of parameters is analyzed. Partial Rank Correlation Coefficient (PRCC) is utilized. The Latin Hypercube Sampling (LHS) technique is used (3). Parameter set is selected from the uniform distribution for each parameter and the output of PRCC is the reproductive number. The PRCC lies between –1 and 1. The sign of the value indicates whether the correlation is positive or negative while the magnitude represents relative importance of the parameter on the reproductive number. The PRCC values for each parameter are listed in Table 4. Parameters with a PRCC significantly (p<0.01) different from zero are indicated with an asterisk (*) in Figure 2 and Table 4.

**Figure 2 PRCC values**

**Table 4 PRCC values**

| Parameter | PRCC value |
| --- | --- |
| $q$ | -0.0373 |
| $\kappa$ | -0.6818* |
| $p$ | -0.1467* |
| $\alpha$ | -0.7038* |
| $\delta$ | 0.0067 |
| $\gamma_{A}$ | 0.0488* |
| $\gamma_{Q}$ | 0.0009 |

*significant ($p$-value <0.01)

**The reproductive number**$\mathbf{and intervention strategies}$

With the parameter listed in the table, the reproductive number is calculated as $\mathcal{R}= \beta/\alpha=4.1192$. In order to decrease $\mathcal{R}$, the transmission rate is reduced through nonpharmaceutical intervention strategy and the quarantine rate needs to be increased. Increasing the quarantine rate means the average period from symptom onset to be quarantined needs to be decreased. Figure 3 depicts a contour map of reproductive number according to reduction of transmission rate and period from symptom onset to quarantine. The colorbar indicates the reproductive number. The x-axis indicates the transmission rate and y-axis represents the averaged period from symptom onset to quarantine. The black thick curve represents the combination of transmission rate and period from symptom onset to quarantine

The red circle at the top right shows the baseline scenario in the model. Note that $\alpha=0.2$ means the average period from symptom onset to quarantine is 5 days. As a nonpharmaceutical intervention is implemented (the transmission rate reduction), the reproductive number moves left. The reproductive number moves down as the quarantine intervention is reinforced.

**Figure 3 A contour map of the reproductive number as nonpharmaceutical and quarantine intervention changes**

References

1. Efron T. Bootstrap Methods for Standard Errors, Confidence Intervals, and Other Measures of Statist. Statistical Science. 1986.

2. Johnson RW. An Introduction to the Bootstrap. Teaching Statistics. 2001;23(2):49-54.

3. Marino S, Hogue IB, Ray CJ, Kirschner DE. A methodology for performing global uncertainty and sensitivity analysis in systems biology. J Theor Biol. 2008;254(1):178-96.
